# Enabling Transfemoral TAVI Through a Hostile Abdominal Aortic Anatomy and a Migrated Mesenteric Stent Fragment: Review of the Literature and a Case Presentation

**DOI:** 10.3390/life16091527

**Published:** 2026-09-14

**Authors:** Catalina-Andreea Parasca, Crina-Ioana Radulescu, Pavel Platon, Andreea Calin, Serban Bubenek-Turconi, Bogdan A. Popescu, Vlad Anton Iliescu

**Affiliations:** 1 Faculty of Medicine, Carol Davila University of Medicine and Pharmacy, 050474 Bucharest, Romaniavlad.iliescu@umfcd.ro (V.A.I.); 2 Department of Cardiovascular Surgery, “Prof. Dr. C.C. Iliescu” Emergency Institute for Cardiovascular Diseases, 022328 Bucharest, Romania; 3 Department of Interventional Cardiology, “Prof. Dr. C.C. Iliescu” Emergency Institute for Cardiovascular Diseases, 022328 Bucharest, Romania; 4 Department of Cardiology, “Prof. Dr. C.C. Iliescu” Emergency Institute for Cardiovascular Diseases, 022328 Bucharest, Romania; 5 Department of Anesthesiology and Intensive Care, “Prof. Dr. C.C. Iliescu” Emergency Institute for Cardiovascular Diseases, 022328 Bucharest, Romania

**Keywords:** transcatheter aortic valve implantation, transfemoral access, hostile abdominal aorta, abdominal aortic stenosis, vascular access facilitation, superior mesenteric artery stent fracture, balloon angioplasty

## Abstract

Hostile abdominal aortic anatomy may limit transfemoral transcatheter aortic valve implantation (TAVI), even when the iliofemoral access vessels are suitable. The principal anatomical substrate is advanced atherosclerotic calcification, which may cause luminal stenosis, reduced arterial compliance, and an increased risk of vascular injury or embolization during large-bore device passage. Evidence regarding endovascular access facilitation is derived predominantly from iliofemoral interventions, whereas experience with direct modification of the abdominal aorta remains limited. This review examines balloon angioplasty, intravascular lithotripsy, and other endovascular strategies used to facilitate transfemoral TAVI in hostile iliofemoral and aortic anatomy, complemented by an illustrative case highlighting the additional challenge posed by intraluminal foreign material. Within this context, we present a 75-year-old high-risk patient with symptomatic severe aortic stenosis and significant coronary artery disease, in whom otherwise suitable iliofemoral access was complicated by focal calcific abdominal aortic stenosis and an asymptomatic impacted fragment of a fractured superior mesenteric artery stent following a previous intervention. A staged strategy comprising coronary revascularization, abdominal aortic balloon angioplasty with apposition of the fragment against the aortic wall, and subsequent balloon-expandable TAVI was successfully performed. This case underscores the importance of individualized, imaging-guided planning for complex transfemoral access.

## 1. Introduction

Transcatheter aortic valve implantation (TAVI) has emerged as a pivotal intervention for patients with severe aortic stenosis, particularly among those at high or prohibitive surgical risk. Among the various access routes for TAVI, the transfemoral (TF) approach is recognized as the preferred method because of its minimally invasive nature and lower complication rates than alternative access routes such as transapical, transaortic, axillary, or subclavian approaches [1]. However, achieving TF access can be challenging in patients with a complex vascular anatomy, such as those with severe atherosclerosis and abdominal aortic stenosis.

Severe abdominal aortic atherosclerosis, often compounded by calcified iliofemoral arteries, poses significant challenges for TF-TAVI. This condition can complicate the passage of the delivery system through the vascular pathway, necessitating advanced interventional techniques to facilitate the procedure. Intravascular lithotripsy (IVL), which uses circumferential pulse sonic pressure waves to fracture vascular calcifications, has been introduced as a promising solution to improve vascular compliance and enable the safe passage of the TAVI delivery system [2].

Recent advancements in imaging tools and planning have significantly enhanced the success rate of TF-TAVI in patients with complex vascular anatomy. Comprehensive preprocedural computed tomography (CT) angiography is essential to assess the extent of vascular calcification, vessel diameter, and tortuosity [3]. This imaging modality allows for meticulous planning and identification of potential obstacles that may impede the TF approach. Additionally, advanced 3D reconstruction techniques and virtual reality simulations are increasingly being used to optimize procedural strategies and anticipate complications [4].

Patients with severe abdominal aortic stenosis due to atherosclerosis present a unique subset of challenges. This condition not only increases the technical difficulty of achieving TF access but also elevates the risk of vascular complications. In such cases, balloon angioplasty or lithotripsy can be employed to dilate the stenotic segments and modify calcifications, thereby facilitating the passage of the TAVI device. The Copenhagen experience with IVL-assisted TF-TAVI has demonstrated promising outcomes, with successful device delivery and minimal vascular complications in a cohort of patients with heavily calcified iliofemoral arteries [2]. Despite these advancements, a significant proportion of patients may still require alternative access routes when TF access is not feasible. These include transaxillary, transcarotid, or trans-subclavian approaches, which, although more invasive, provide viable options for TAVI delivery in patients with contraindications to the TF route. Each alternative access route has its own set of technical considerations and potential complications, necessitating a tailored approach based on the individual patient anatomy and comorbidities. However, achieving TF access in patients with complex vascular anatomy, such as those with severe atherosclerosis and abdominal aortic stenosis, requires advanced imaging techniques, meticulous planning, and innovative interventional strategies [5]. As this field continues to evolve, ongoing research and technological advancements will further enhance the safety and efficacy of TAVI in this challenging patient population.

## 2. Case Report

### 2.1. History and Presentation

A 75-year-old female patient presented to our hospital with progressive dyspnea, orthopnea, and angina on mild exertion at admission. The patient’s medical history included systemic hypertension grade III, dyslipidemia, type 2 diabetes mellitus (non-insulin-dependent), and severe systemic atherosclerosis. Three years prior to this admission, the patient underwent major abdominal surgery in a different medical center for mesenteric ischemia, during which right hemicolectomy and superior mesenteric artery (SMA) angioplasty and stenting were performed. Additionally, she was diagnosed with chronic kidney disease (CKD) class IIIA, moderate chronic anemia, hypothyroidism, chronic obstructive pulmonary disease (COPD) GOLD II, and bilateral gonarthrosis with poor mobility.

### 2.2. Clinical Findings

Upon clinical examination, the patient exhibited hypertension with a blood pressure of 150/75 mmHg and a heart rate of 80 beats per minute, rhythmic heart sounds, and a grade 4/6 aortic systolic murmur. Electrocardiogram (ECG) showed sinus rhythm with electrical criteria indicative of left ventricular hypertrophy and ST-segment and T-wave abnormalities in the precordial leads. Laboratory investigations revealed an elevated N-terminal pro–B-type natriuretic peptide (NT-proBNP) level of 3606 pg/mL, moderate chronic anemia with functional iron deficiency (hemoglobin, 9.4 g/dL; serum iron, 47 µg/dL; ferritin, 171.9 ng/mL; transferrin saturation, 7.9%), and impaired renal function (serum creatinine, 1.35 mg/dL; urea, 79 mg/dL; estimated glomerular filtration rate [eGFR], 45 mL/min/1.73 m^2^). The lipid profile showed marked dyslipidemia (total cholesterol, 339 mg/dL; LDL cholesterol, 251 mg/dL; HDL cholesterol, 45 mg/dL). Thyroid function tests were within normal limits under thyroid hormone replacement therapy.

### 2.3. Imaging and Diagnostic Workup

**Figure 1:**
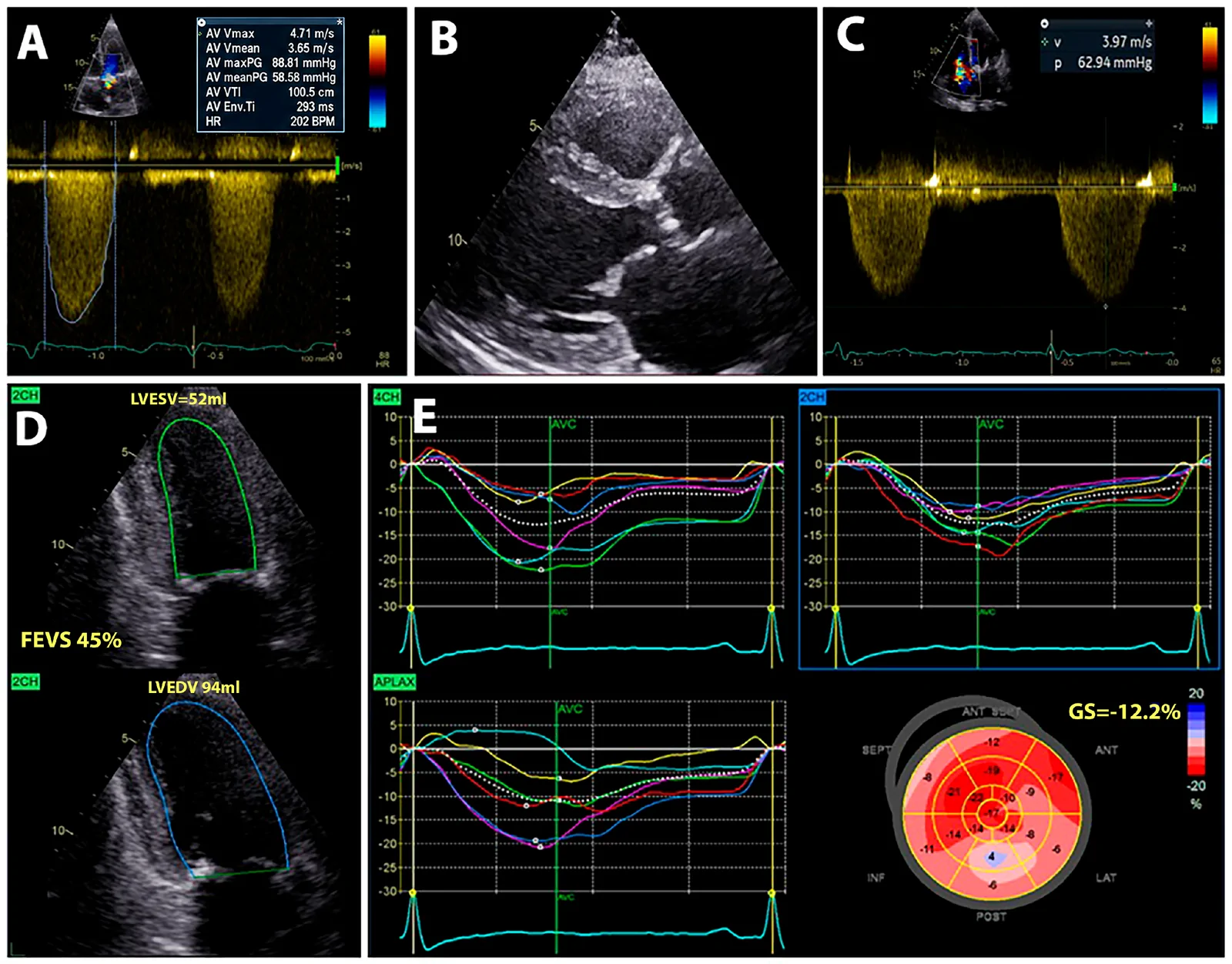
Transthoracic echocardiographic evaluation of aortic valve and cardiac function. (**A**) Continuous-wave Doppler assessment of aortic valve stenosis. (**B**) Aortic valve aspect (PLAX view). (**C**) Severe pulmonary hypertension. (**D**,**E**) Myocardial function assessment using LVEF and LV longitudinal strain.

Transthoracic echocardiography revealed a severely calcified tricuspid aortic valve, with restricted cusp motion and an indexed AVA of 0.33 cm^2^/m^2^ and a peak/mean transvalvular gradient of 88/58 mmHg. Additionally, mild left ventricular systolic dysfunction was noted (LVEF 45%), with impaired LV relaxation, mild mitral regurgitation, moderate tricuspid regurgitation, and severe pulmonary hypertension with an estimated PASP of 60 mmHg (Figure 1). 

**Figure 2:**
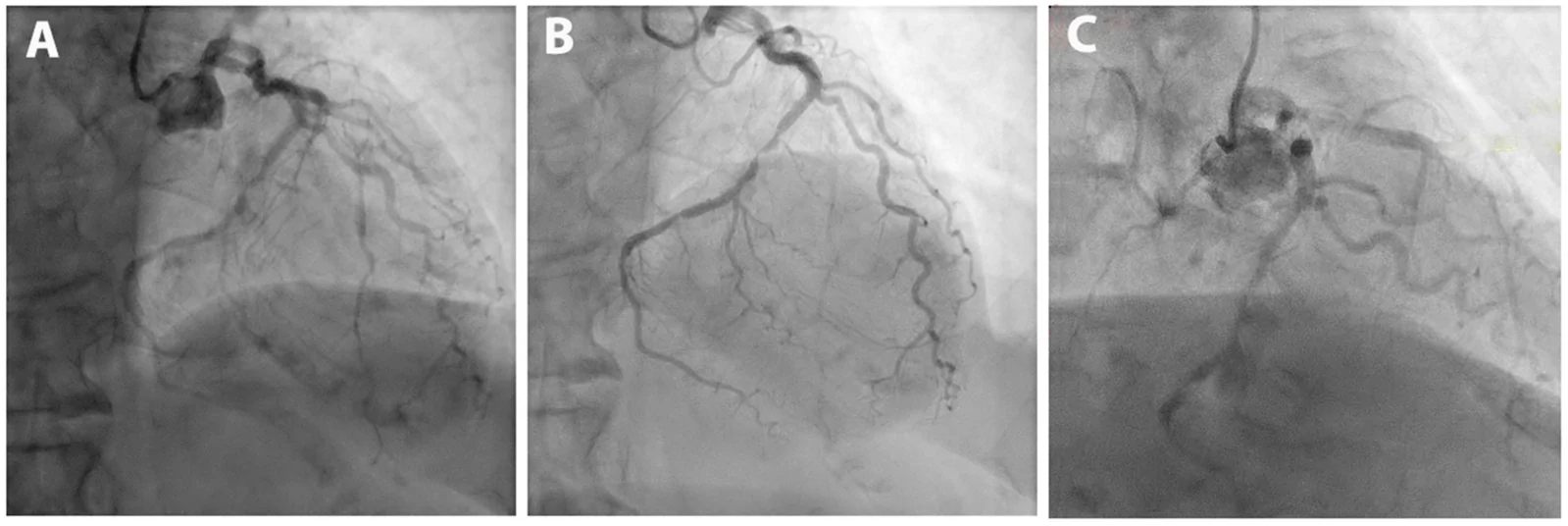
Coronary angiography revealing two-vessel coronary artery disease with LAD subocclusion (**A**), 90% stenosis of LCx (**B**), and hypoplastic RCA (**C**).

As part of the institutional pre-TAVI evaluation protocol in use at the time and given the patient’s exertional angina and the high likelihood of concomitant coronary artery disease, invasive coronary angiography was performed to define the coronary anatomy and determine the need for revascularization before TAVI. Coronary angiography was initially attempted via radial access; however, marked vascular tortuosity prevented adequate catheter advancement, necessitating conversion to femoral access. Coronary angiography revealed severe two-vessel coronary artery disease, with a subocclusive proximal left anterior descending artery (LAD) lesion and severe proximal left circumflex artery (LCx) stenosis, in the presence of a hypoplastic right coronary artery (RCA) (Figure 2).

**Figure 3:**
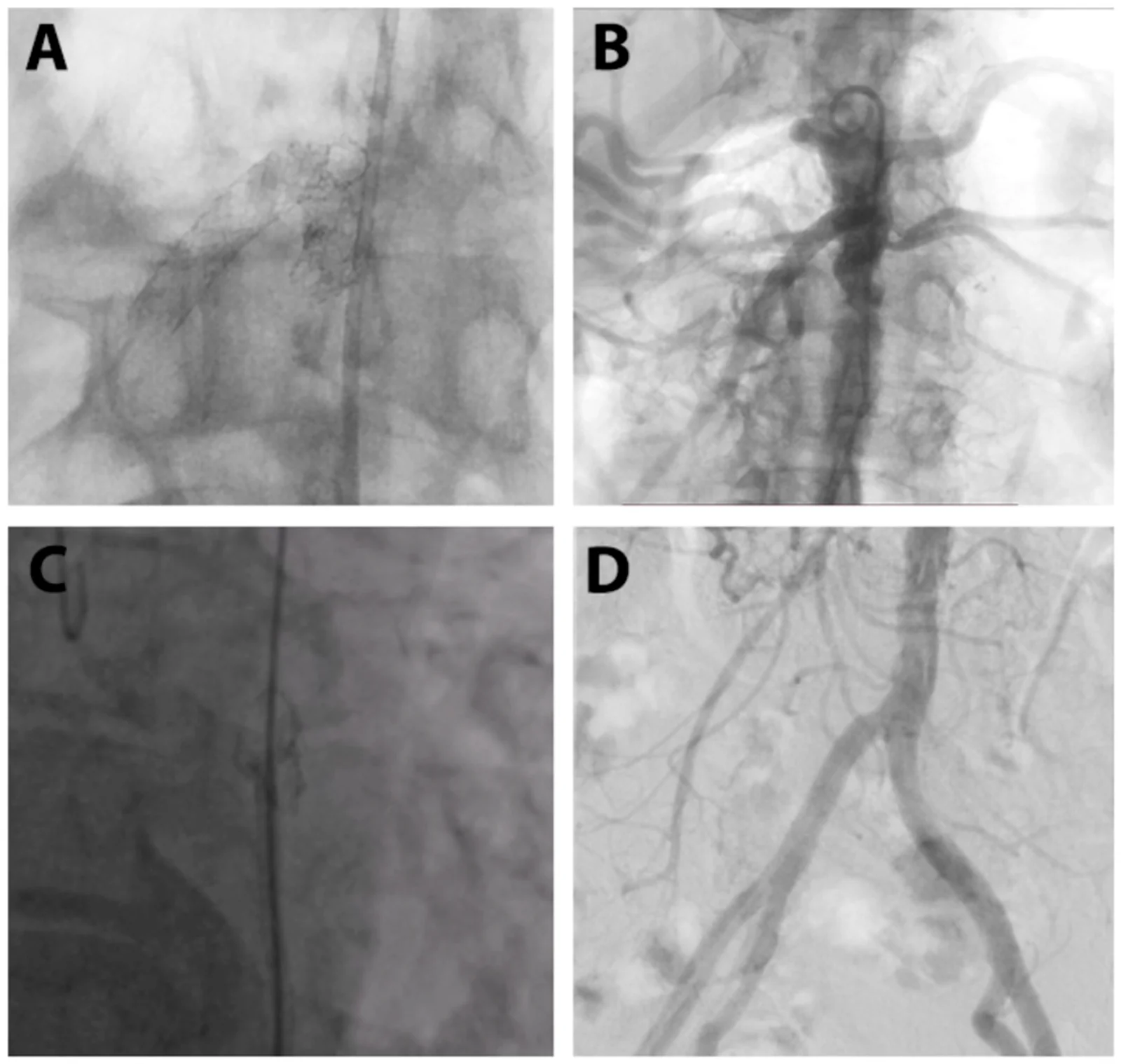
Aortography revealed: (**A**) severe atherosclerotic disease of the abdominal aorta with moderate infrarenal aortic stenosis; (**B**) patent superior mesenteric artery stent; (**C**) mobile stent fragment; (**D**) no significant iliac artery stenosis.

During femoral catheter advancement, unexpected resistance was encountered at the level of the abdominal aorta. Aortography demonstrated extensive atherosclerotic disease with focal infrarenal stenosis and an unexpected migrated stent fragment from the previous SMA intervention. During pigtail catheter passage, the fragment exhibited cranio-caudal movement, confirming its mobility. Dedicated aortography further demonstrated a patent SMA with the residual stent remaining in situ and no significant iliac artery stenosis (Figure 3).

Evaluation of the femoral arteries was also performed using Doppler ultrasound, which revealed a normal triphasic waveform with slightly reduced flow velocities, adequate vessel diameter, and minimal calcification on the posterior wall. 

**Figure 4:**
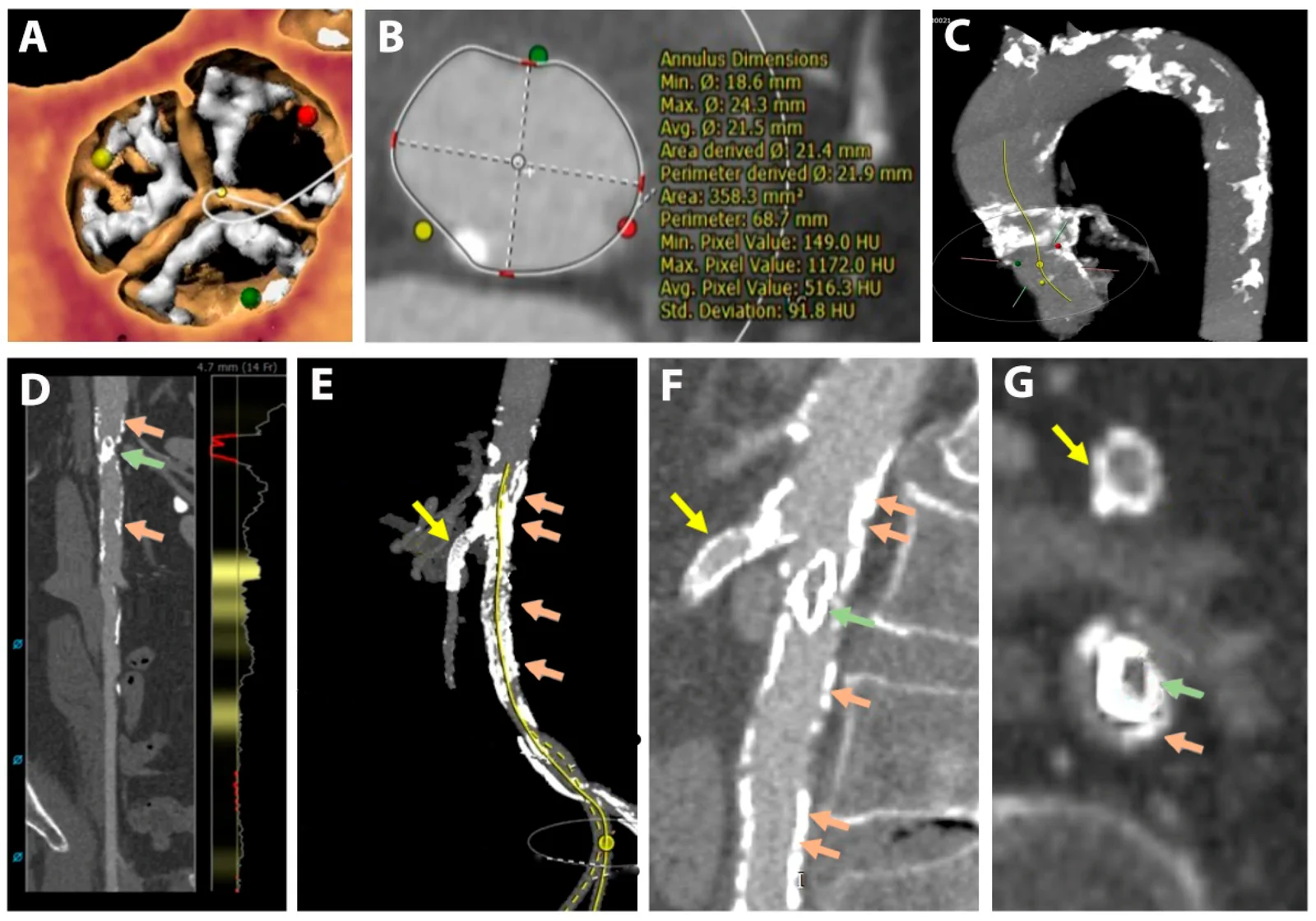
Preprocedural planning computed tomography. (**A**) Three-dimensional reconstruction of the heavily calcified tricuspid aortic valve. (**B**) Aortic annular measurements compatible with implantation of a 23-mm balloon-expandable valve. (**C**) Multiplanar reconstruction of LVOT, STJ, aortic arch and descending thoracic aorta calcification. (**D**) Assessment of the vascular access route demonstrating extensive calcific atherosclerosis of the abdominal aorta with focal infrarenal aortic stenosis and migrated stent fragment; the iliofemoral vessels were considered suitable for transfemoral access. (**E**) Multiplanar reconstruction demonstrating the calcified abdominal aorta and in situ SMA stent. (**F**) Close-up sagittal view demonstrating the in situ SMA stent and migrated stent fragment within the abdominal aorta, and extensive abdominal aortic calcification. (**G**) Axial CT images demonstrating the in situ SMA stent and the migrated stent fragment within the abdominal aortic lumen. Stent fragment—green arrow; SMA and residual stent—yellow arrow; aortic calcifications—orange arrow. Abbreviations: SMA, superior mesenteric artery; STJ, sinotubular junction; LVOT, left ventricular outflow tract.

As per the institutional TAVI protocol, contrast-enhanced ECG-gated CT angiography (CTA) was performed for comprehensive assessment of the aortic valve, aortic root, aorta, and iliofemoral access vessels (Figure 4). CTA demonstrated a tricuspid calcified aortic valve with mild left ventricular outflow tract calcification and an annular area of 358 mm^2^, suitable for a 23-mm balloon-expandable valve. The abdominal aorta showed extensive calcific atherosclerotic disease extending from the visceral branches to the aortic bifurcation, with a focal stenosis located 7 mm below the inferior margin of the SMA ostium and 3 mm below the lowest renal artery (left renal artery). At the stenotic segment, the minimum luminal dimensions were 7 × 8 mm, compared with proximal and distal reference dimensions of 20 × 19 mm and 16 × 17 mm, respectively, corresponding to approximately 55–62% diameter stenosis depending on the reference segment used. An 18-mm-long migrated stent fragment, measuring approximately 6 × 7 mm in transverse dimensions, was oriented longitudinally within the aortic lumen at this level. The fragment was non-occlusive, with its proximal extent approximately 10 mm below the superior margin of the SMA ostium, and entirely below the renal arteries. Detailed procedural data regarding the type, dimensions, and implantation technique of the previously implanted SMA stent were unavailable, as the original records could not be retrieved from the referring institution. The iliofemoral vessels were non-tortuous and demonstrated only mild, predominantly posterior-wall calcification. On the right, the common femoral, external iliac, and common iliac arteries measured 5.6 × 8.2 mm, 5.0 × 6.4 mm, and 7.2 × 8.5 mm at their narrowest segments, respectively; corresponding measurements on the left were 5.5 × 6.1 mm, 5.0 × 5.8 mm, and 7.0 × 8.1 mm. Thus, although the iliofemoral anatomy itself was considered suitable for transfemoral access, passage of the large-bore delivery system was considered unsafe because of the combination of the severely calcified abdominal aorta, focal aortic stenosis, and intraluminal migrated stent fragment. Regarding the abdominal viscera, no additional significant abnormalities were identified apart from bilateral renal cortical thinning and mild cortical scarring.

### 2.4. Risk Assessment and Treatment Strategy

Assessment of operative risk yielded an STS Predicted Risk of Mortality score of 12.13% and a EuroSCORE II of 25.96%, indicating a high surgical risk. Frailty assessment using the Essential Frailty Toolset (EFT) yielded a score of 3/5, based on a prolonged five-chair-rise time (>15 s; 1 point), cognitive impairment (MMSE < 24; 1 point), anemia (hemoglobin 9.5 g/dL; 1 point), and a normal serum albumin level (0 points). Given the high surgical risk and concomitant frailty, the Heart Team proposed a staged percutaneous treatment strategy: (i) myocardial revascularization via percutaneous coronary intervention (PCI), (ii) management of the abdominal aorta to enable TF access, and (iii) transfemoral TAVI. PCI was prioritized because of the patient’s exertional angina and the severity and proximal location of the LAD and LCx lesions. Subsequent transfemoral TAVI was considered feasible provided that the abdominal aortic obstruction, resulting from the combination of atherosclerotic stenosis and the migrated mesenteric stent fragment, could be safely overcome. Medical therapy was concurrently optimized according to current guidelines, including neurohormonal blockade for heart failure, high-dose statins for dyslipidemia, and intravenous ferric carboxymaltose for iron-deficiency anemia. 

### 2.5. Staged Treatment

Following multidisciplinary Heart Team evaluation, a staged percutaneous strategy was selected, with the aim of preserving the transfemoral route, as alternative non-femoral access was considered to carry a higher periprocedural risk. The treatment sequence comprised coronary revascularization, endovascular preparation of the abdominal aorta, and subsequent transfemoral TAVI. Given the severe proximal two-vessel coronary artery disease and associated ischemic symptom burden, coronary revascularization was considered the first therapeutic priority before proceeding with aortic access preparation and TAVI. The abdominal aortic lesion was subsequently addressed as a separate procedure to establish a safe route for large-bore transfemoral access. Given the severely atherosclerotic and stenotic aorta and the presence of a mobile embolized stent fragment, concomitant aortic angioplasty and TAVI were considered to entail an increased risk of aortic dissection or rupture, as well as further fragment migration during manipulation and advancement of the large-bore delivery system. A staged approach allowed the angiographic result of aortic angioplasty and stable apposition of the stent fragment against the aortic wall below the origin of the superior mesenteric artery to be confirmed before proceeding with TAVI. Furthermore, in the setting of stage IIIA chronic kidney disease, separating the interventions allowed the contrast burden of each procedural session to be limited, while also avoiding the prolonged duration and technical complexity of a single combined coronary, peripheral, and structural intervention. 

As the first stage of the planned treatment strategy, PCI was performed one week after the initial diagnostic coronary angiography because of the patient’s exertional angina and severe proximal two-vessel coronary artery disease. The procedure was undertaken in the cardiac catheterization laboratory via femoral arterial access under local anesthesia. Two drug-eluting stents were successfully implanted in the proximal LAD and LCx, achieving complete coronary revascularization without procedural complications. Post-PCI antithrombotic therapy consisted of aspirin 100 mg and clopidogrel 75 mg once daily, which were continued throughout the subsequent staged interventions in accordance with guideline recommendations. 

**Figure 5:**
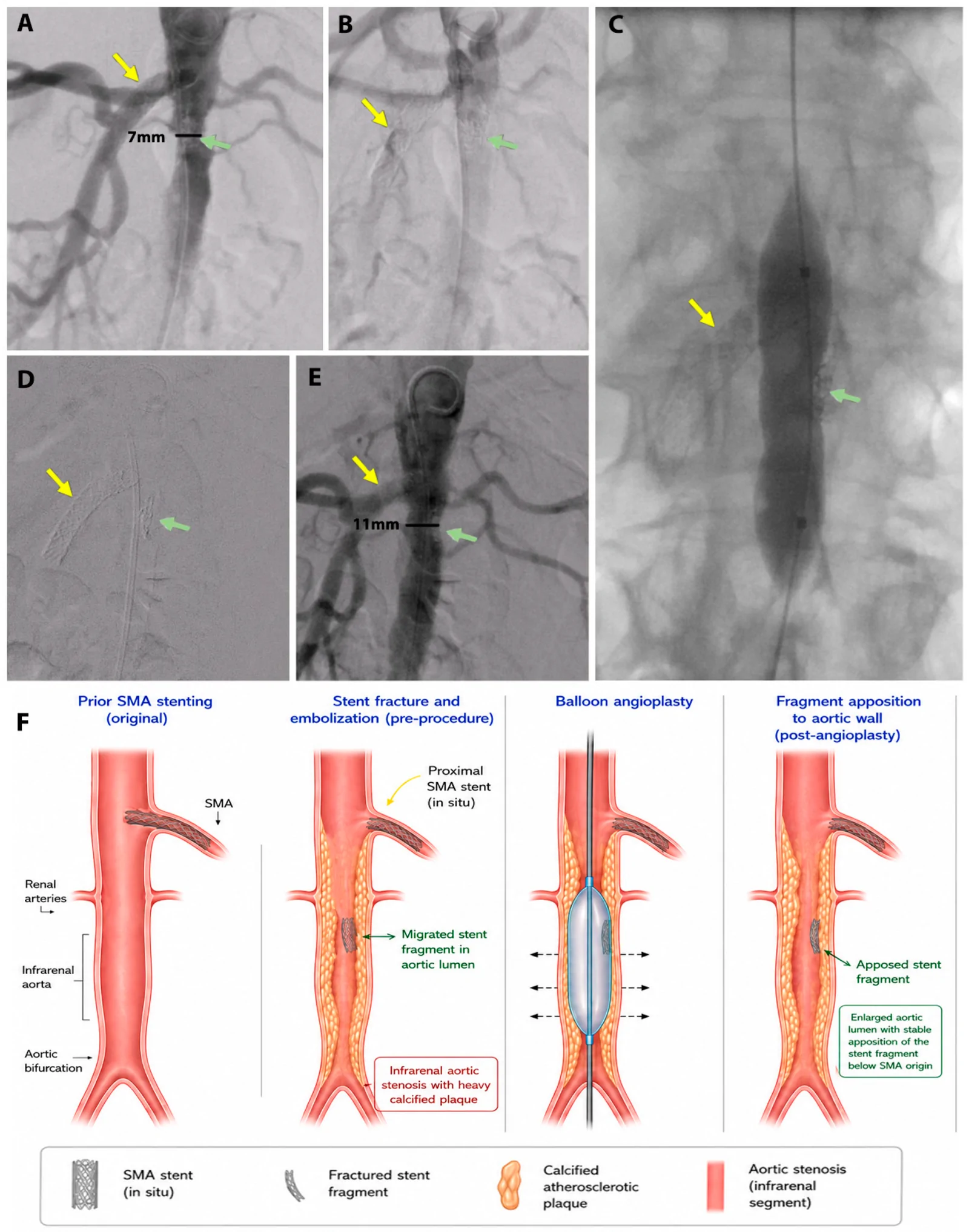
Endovascular preparation of the abdominal aorta. (**A**,**B**) Aortography demonstrating infrarenal aortic stenosis (7 × 8 mm), the in situ SMA stent (yellow arrows), and the migrated stent fragment (green arrows); (**C**) balloon angioplasty with a 12 × 40-mm VACS^®^ II balloon, simultaneously enlarging the stenotic aortic segment and displacing the fragment toward the aortic wall; (**D**) stent fragment apposed to the aortic wall after angioplasty; (**E**) final aortography demonstrating an enlarged aortic lumen (11 × 12 mm) and stable fragment apposition; (**F**) schematic illustration of balloon angioplasty and fragment apposition. Abbreviation: SMA, superior mesenteric artery.

Four weeks after PCI, the second intervention was performed in the cardiac catheterization laboratory under local anesthesia via right femoral arterial access. The contralateral left common femoral artery was reserved for secondary access and potential delivery of a BeGraft Aortic balloon-expandable covered stent (Bentley InnoMed GmbH, Hechingen, Germany) in the event of aortic rupture or flow-limiting dissection. Intraprocedural anticoagulation was achieved with intravenous unfractionated heparin (7000 IU; approximately 100 IU/kg), with a target activated clotting time (ACT) of 200–250 s. A hydrophilic Radifocus™ Guidewire M (Terumo Corporation, Tokyo, Japan) was carefully advanced through the stenotic abdominal aortic segment, passing between the migrated stent fragment and the aortic wall rather than through the stent struts, as confirmed by fluoroscopy in multiple projections. The hydrophilic guidewire was subsequently exchanged for a Hi-Torque Supra Core™ guidewire (Abbott, Abbott Park, IL, USA) to provide additional support for the intervention. Preprocedural imaging demonstrated a minimum aortic luminal diameter of approximately 7 × 8 mm at the level of the infrarenal aortic stenosis, with the residual lumen further compromised by the mobile stent fragment. Progressive balloon angioplasty was performed using 8-, 10-, and 12-mm × 40-mm VACS^®^ II balloons (OSYPKA, Rheinfelden, Germany), inflated to 4, 8, and 10 atm, respectively (Figure 5). This stepwise approach simultaneously enlarged the stenotic aortic lumen and progressively displaced and apposed the migrated stent fragment against the aortic wall, below the origin of the superior mesenteric artery. Final angiography demonstrated an increase in the minimum aortic luminal dimensions from 7 × 8 mm to approximately 11 × 12 mm, without evidence of aortic rupture or flow-limiting dissection. The stent fragment remained securely apposed to the aortic wall below the SMA origin and renal arteries, without compromising visceral vessel perfusion. No postprocedural CT examination was performed.

**Figure 6:**
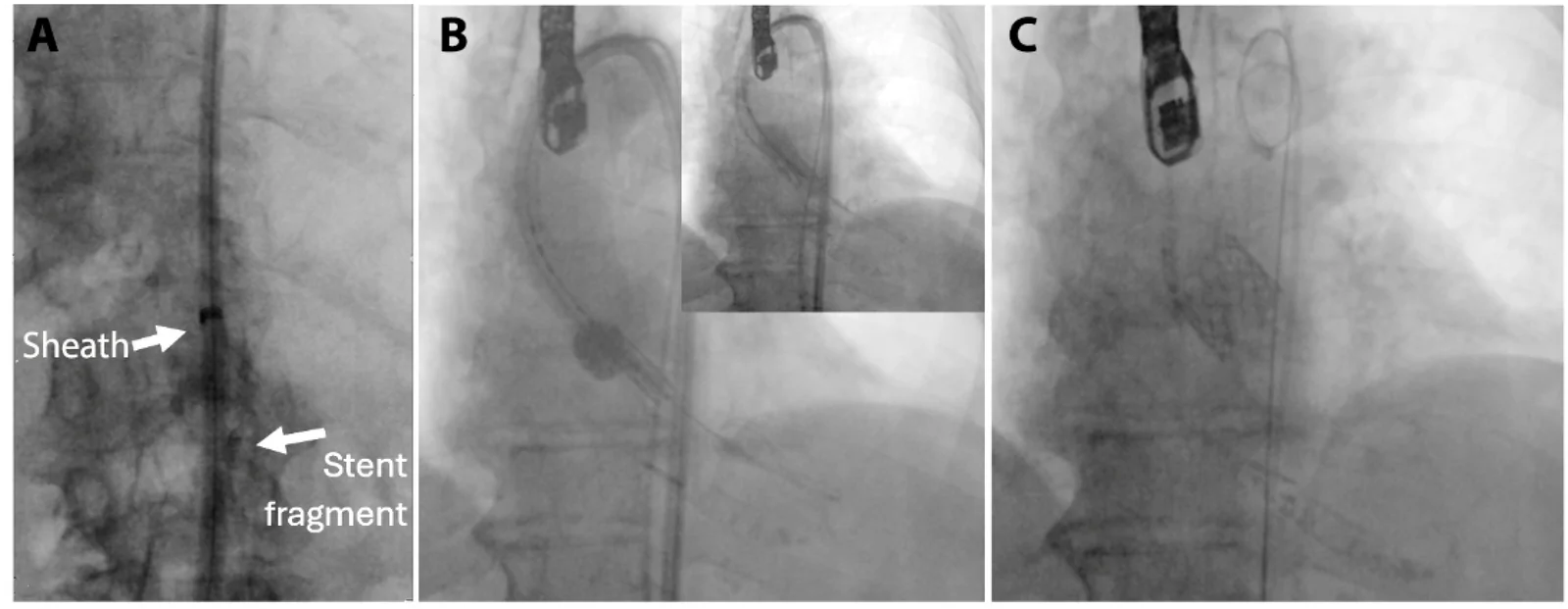
Transfemoral TAVI procedure. (**A**) Advancement of the 14-Fr expandable introducer sheath through the previously treated abdominal aortic segment and past the stent fragment apposed to the aortic wall; (**B**) positioning and deployment of the 23-mm balloon-expandable transcatheter aortic valve; (**C**) final aortographic assessment following valve implantation.

**Figure 7:**
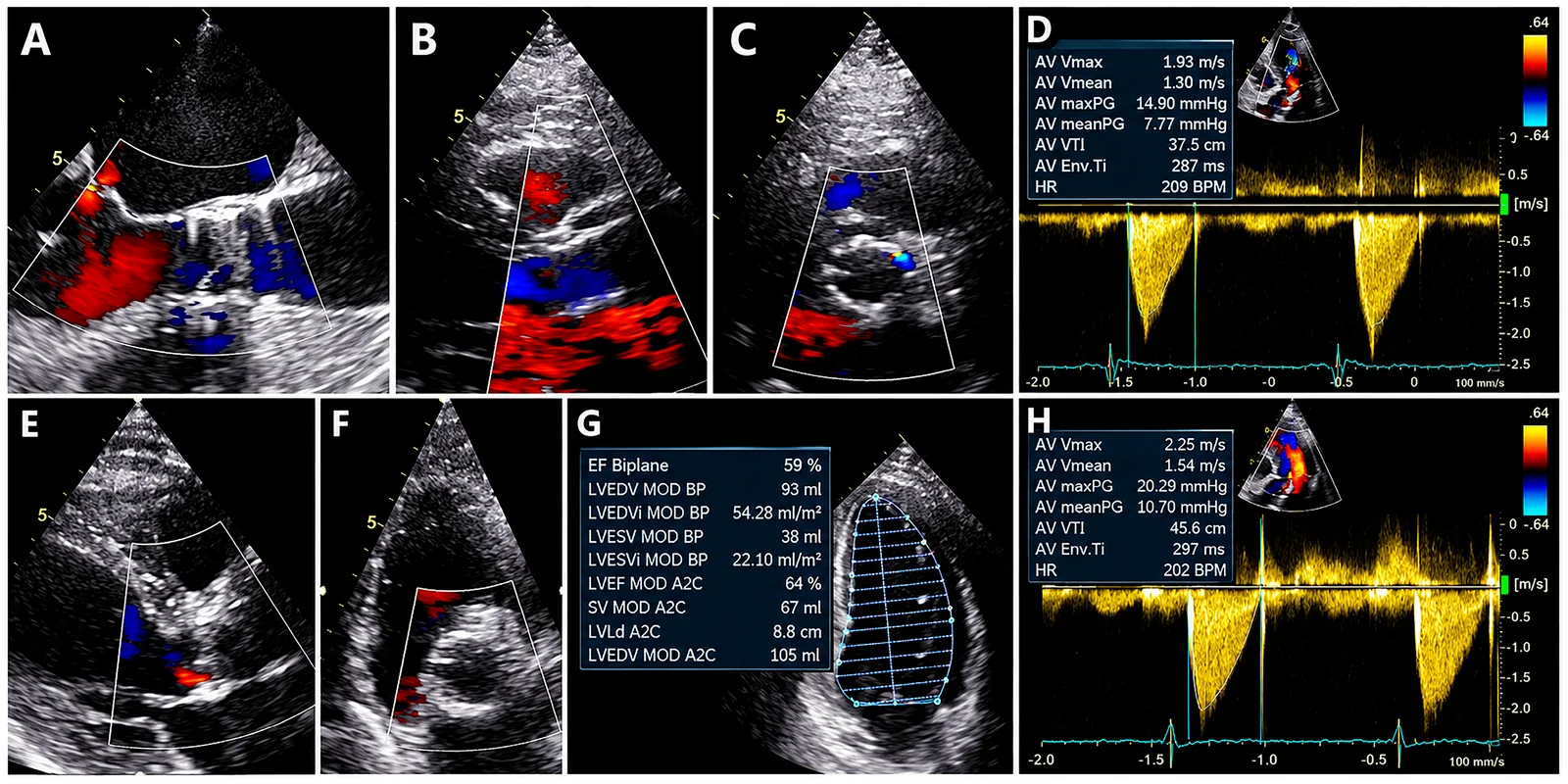
Echocardiographic assessment after TAVI: (**A**–**C**). Intraprocedural transesophageal echocardiography following valve implantation, demonstrating appropriate prosthetic valve position and function with trivial paravalvular regurgitation; (**D**) transthoracic Doppler echocardiography at 1-month follow-up demonstrating preserved prosthetic valve hemodynamics; (**E**–**H**) transthoracic echocardiographic assessment at 2-year follow-up, demonstrating preserved left ventricular systolic function and stable transcatheter valve function with low transprosthetic gradients.

Two weeks after successful preparation of the abdominal aortic access route, the patient underwent the third and final intervention. Transfemoral TAVI was performed in the hybrid operating room, in accordance with institutional protocol, under conscious sedation and invasive hemodynamic monitoring. Following placement of a SAFARI^2^™ guidewire (Boston Scientific Corporation, Marlborough, MA, USA), a 14-Fr eSheath™ expandable introducer sheath (Edwards Lifesciences, Irvine, CA, USA) was carefully advanced through the previously treated abdominal aortic segment and past the apposed stent fragment without fragment displacement or evidence of aortic injury. A 23-mm Edwards SAPIEN 3 Ultra transcatheter heart valve (Edwards Lifesciences, Irvine, CA, USA) was subsequently advanced through the sheath and successfully implanted via the right transfemoral approach, and access-site hemostasis was achieved using a pre-closed percutaneous suture-mediated closure device (Figure 6). Final angiography and echocardiography confirmed optimal prosthesis position and function, with only trivial residual regurgitation (Figure 7). With contrast exposure minimized and carefully monitored periprocedural intravenous hydration administered throughout the staged treatment, renal function remained stable, without acute kidney injury. Exact contrast volumes could not be retrieved retrospectively. The postprocedural course was uneventful, and the patient was discharged two days after TAVI.

### 2.6. Follow-Up

At 1-month follow-up, the patient demonstrated significant clinical and functional improvement, accompanied by recovery of left ventricular systolic function (LVEF from 45% to 60%) and absence of recurrent angina. NT-proBNP levels decreased (from 3606 to 1825 pg/mL) and hemoglobin levels increased (from 9.4 to 10.3 g/dL), whereas pre-existing renal dysfunction persisted (serum creatinine of 1.37 mg/dL and an eGFR of 41 mL/min/1.73 m^2^). At 2-year follow-up, the patient remained clinically stable in NYHA functional class II, without recurrent angina or symptoms suggestive of mesenteric or lower-limb ischemia, including postprandial abdominal pain, weight loss, or claudication, and body weight remained stable. Transthoracic echocardiography demonstrated preserved left ventricular systolic function (LVEF 60%), trivial paravalvular regurgitation and stable prosthetic valve hemodynamics (peak and mean transprosthetic gradients of 20 and 10 mmHg, respectively). Biochemical follow-up paralleled the sustained clinical improvement, with NT-proBNP decreasing to 1256 pg/mL and hemoglobin increasing to 11.5 g/dL, while renal function remained stable compared with baseline (eGFR 45 mL/min/1.73 m^2^). No dedicated vascular imaging of the abdominal aorta or SMA was performed during long-term follow-up. Overall, the sustained clinical improvement and stable prosthetic valve performance support the favorable outcome of the carefully planned and staged interventional strategy.

## 3. Discussion and Review of Literature

The present case illustrates a uniquely challenging scenario in contemporary structural heart disease practice: the convergence of severe symptomatic aortic stenosis, severe atherosclerotic abdominal aortic disease, and an unexpected intraluminal stent fragment from a prior mesenteric intervention, all of which rendered transfemoral access initially unsafe without prior endovascular preparation. Through a staged, multidisciplinary approach employing balloon angioplasty to both reposition the migrated stent fragment and dilate the stenotic aortic segment, we successfully performed TF-TAVI, avoiding the higher morbidity of alternative access, and achieving durable clinical improvement up to two years of follow-up. This discussion integrates the unique features of our case with the available evidence from the literature. 

### 3.1. Transfemoral vs. Non-Transfemoral

Transfemoral access is the preferred approach for TAVI when anatomically feasible and is supported by guideline recommendations and extensive observational evidence [6,7]. Systematic evaluation of transfemoral access, followed by subclavian/axillary, carotid, transcaval, and transapical access is recommended to achieve optimal results post-TAVI according to 2025 ESC/EACTS Guidelines [6]. Despite the strong recommendations for TF access, a subset of patients will inevitably require alternative access to achieve transcatheter treatment of SAS. When transfemoral access is not feasible, surgical aortic valve replacement is favored over non-transfemoral TAVI in low-risk patients, whereas alternative access should be considered in high- or prohibitive-risk patients [6]. 

Among non-transfemoral approaches, transaxillary/subclavian and transcarotid access are currently the preferred alternatives, offering high procedural success and acceptable early outcomes [8,9,10,11]. Nevertheless, observational studies and meta-analyses have demonstrated higher long-term mortality and increased vascular complications compared with transfemoral TAVI [12]. In contrast, transapical and transaortic access are associated with greater procedural invasiveness and significantly higher rates of in-hospital and 1-year mortality, bleeding, and perioperative complications, leading to a marked decline in their use [13,14]. Transcaval access remains a valuable option in carefully selected patients but requires favorable anatomy and specialized expertise [15,16]. Evidence from contemporary registries and meta-analyses demonstrates that the disadvantage of non-transfemoral access extends beyond procedural morbidity, translating into increased long-term mortality. Therefore, whenever anatomically feasible, transfemoral access should be prioritized, with lesion-specific endovascular optimization—including balloon angioplasty, intravascular lithotripsy, or selective stent/stent-graft implantation—to facilitate safe large-bore device delivery [13].

The transfemoral approach was favored in our case despite the mechanical challenges, because of the patient’s high surgical risk, concomitant frailty, multiple comorbidities, and suitable iliofemoral anatomy. As the abdominal aortic findings represented the only obstacle, preprocedural imaging suggested that the impacted stent fragment could be safely displaced by balloon angioplasty in a staged pre-TAVI procedure, allowing for the less invasive TF route. This strategy was consistent with the established preference for transfemoral access when it can be achieved safely, while avoiding the need for alternative access and achieving sustained clinical improvement over two years of follow-up [13].

### 3.2. Mesenteric Artery Stent Fracture and Migration

Stent fracture and migration are known complications of endovascular interventions in the peripheral and visceral arterial territories [17], yet their occurrence in the superior mesenteric artery remains sparsely reported [18,19,20]. The SMA is subject to unique biomechanical stress due to diaphragmatic movement, which may predispose implanted stents to mechanical fatigue and eventual fracture, especially when the proximal stent extends into the aortic lumen. The proposed mechanism of fracture involves cyclical compression and torsion of the stent during respiratory excursions, compounded by the fixed angulation of the SMA at its origin from the aorta, which creates a hinge point for mechanical stress concentration. 

A literature review was conducted using the PubMed database to identify published reports of SMA stent fracture and its clinical implications. The search included articles published from database inception through June 2026 using the terms “superior mesenteric artery” AND “stent fracture”, yielding 27 articles. Given the limited evidence, the search was supplemented by screening reference lists, Google Scholar, and relevant journal websites to identify reports not indexed in PubMed. Relevant articles were identified through manual screening of the reference lists of eligible publications. Eligible publications were full-text articles reporting SMA stent fracture, including its clinical presentation, mechanism, management, or outcomes. Articles not related to SMA stent fracture, non-English publications, conference abstracts without full text, editorials, letters, and commentaries were excluded. Therefore 7 articles were included [18,19,20,21,22,23,24], including one non-PubMed-indexed case report [23], and reviewed to summarize the reported mechanisms, clinical presentation, therapeutic approaches, and outcomes of SMA stent fracture, summarized in Table 1. 

The review of the literature identified only 10 reported cases of superior mesenteric artery (SMA) stent fracture (7 reported articles). Most occurred following endovascular treatment for chronic mesenteric ischemia (70%, 7/10), with fewer cases reported after fenestrated endovascular aortic repair (20%, 2/10) and superior mesenteric artery aneurysm stenting (10%, 1/10). Recurrent mesenteric ischemic symptoms were the most common presentation (70%, 7/10), whereas 20% (2/10) of fractures were detected incidentally in asymptomatic patients and one patient (10%) presented with persistent postprandial discomfort. When reported, the interval to fracture ranged from 1 month to 2 years. Stent fracture most frequently resulted in restenosis (50%, 5/10), while stent displacement or embolization occurred in 30% (3/10); the remaining two cases had other or incompletely specified fracture patterns. Endovascular re-intervention with repeat stenting was the predominant treatment (60%, 6/10), surgical repair was required in one case (10%, 1/10), and management was not specified in three cases (30%, 3/10). All cases with reported treatment and outcome data were successfully managed.

It should be acknowledged that the diagnostic pathway in this case reflects the institutional pre-TAVI work-up protocol in use at the time, which included routine invasive coronary angiography. Contemporary TAVI assessment increasingly relies on comprehensive ECG-gated cardiac CT combined with CT angiography of the aorta and iliofemoral access vessels, with invasive coronary angiography used more selectively [6]. Such a CT-based strategy would likely have identified the hostile abdominal aortic anatomy and the migrated stent fragment and might also have detected the proximal coronary lesions, although the accuracy of coronary stenosis assessment may be limited in patients with extensive coronary calcification. Importantly, in the present case, fluoroscopy during femoral catheter advancement provided additional dynamic information by demonstrating cranio-caudal movement of the stent fragment upon contact with the pigtail catheter, prompting dedicated aortography and highlighting its mechanical instability.

The most likely mechanism involved was the progressive stent fatigue with complete fracture, followed by embolization of the detached fragment and distal migration until it became impacted at the level of the stenotic and calcified aortic segment [25]. Although SMA stent fracture has been associated with restenosis, occlusion, and recurrent mesenteric ischemia [18,19,20,21], the fracture in our patient remained clinically silent, with preserved SMA patency and no need for mesenteric reintervention. Its only clinical consequence was the creation of an unexpected mechanical obstacle to transfemoral TAVI, successfully managed through comprehensive CT-based procedural planning and a tailored endovascular approach by balloon angioplasty. While adjunctive endovascular therapies, including intravascular lithotripsy or implantation of an aortic stent or stent graft, may be appropriate in selected patients with severe calcific or obstructive aortic disease [26,27,28,29], careful Heart Team evaluation integrating multimodality imaging and procedural risk assessment supported a less invasive balloon-only strategy in our patient. Balloon angioplasty was considered sufficient to restore an adequate luminal diameter for safe transfemoral TAVI while avoiding additional endovascular implants. The procedure was performed with awareness of the potential risks of aortic rupture, dissection, and distal calcium embolization [30], and a BeGraft Aortic balloon-expandable covered stent (Bentley InnoMed GmbH, Hechingen, Germany) was kept on standby as a bailout option but was ultimately not required. Intravascular lithotripsy was not available at our institution at the time.

### 3.3. TAVI and Hostile Abdominal Aorta

Increasing experience with transfemoral (TF) TAVI in patients with hostile aortoiliac anatomy has expanded the role of endovascular access optimization. Balloon angioplasty, intravascular lithotripsy (IVL), and selective stent or stent-graft implantation have all been successfully employed to treat calcified or stenotic vascular segments, thereby facilitating large-bore sheath delivery while preserving the benefits of the transfemoral approach [13]. Among these techniques, IVL has emerged as an effective strategy for heavily calcified lesions that are poorly compliant to conventional balloon angioplasty. 

Its application has recently been extended to the abdominal aorta, including patients with coral reef aorta or severe calcific aortic stenosis, where IVL-assisted angioplasty has enabled successful TF-TAVI without major vascular complications [26,28,31]. Morris et al. described the first successful use of IVL-assisted balloon angioplasty of a coral reef aorta to facilitate TF-TAVI, demonstrating that even extensive calcific disease of the abdominal aorta can be safely managed with this technique [26]. A recent case report has shown that IVL of a negatively remodeled calcified abdominal aorta can be performed with stepwise low-pressure inflations, achieving adequate luminal expansion for an 18-French sheath with same-day discharge [31]. 

For the specific analysis of endovascular techniques employed to facilitate transfemoral access in patients with hostile aortoiliac anatomy, a further focused literature search was conducted in PubMed from January 2016 through June 2026 using the search terms: (“transcatheter aortic valve implantation” OR “TAVI” OR “TAVR”) AND (“intravascular lithotripsy” OR “balloon angioplasty” OR “peripheral vascular intervention”) AND (“femoral” OR “iliac” OR “iliofemoral” OR “abdominal aorta” OR “aortic calcification” OR “coral reef aorta” OR “hostile access”) AND (“transfemoral”), yielding 43 articles. Relevant articles were identified through manual screening of the reference lists of eligible publications, according to the previously described inclusion and exclusion criteria relevant to the topic. A total of 20 studies were identified, 12 studies describing interventions on the iliofemoral segment [2,32,33,34,35,36,37,38,39,40,41,42] and 8 studies describing interventions on the abdominal aorta [26,28,31,43,44,45,46,47], summarized in Table 2. 

Evidence for iliofemoral access facilitation has evolved from preparatory balloon angioplasty to intravascular lithotripsy (IVL), with contemporary series and registries reporting high rates of successful transfemoral TAVI and acceptable vascular complication rates [2,32,33]. However, these procedures are performed in patients with more advanced peripheral vascular disease and are not without additional risk. In a propensity-matched analysis, Imran et al. reported a higher incidence of the primary composite outcome after IVL-assisted TAVI than after conventional transfemoral TAVI (21.9% vs. 13.7%, *p* < 0.001), driven by higher rates of vascular complications, surgical vascular intervention, and major bleeding [42]. These findings emphasize the importance of careful patient selection when conventional transfemoral access is not feasible or is considered high risk. Comparative studies further suggest that preserving transfemoral access through peripheral vascular intervention may be associated with favorable outcomes compared with alternative access strategies [35,36,41], while the Hostile TAVI Registry supports IVL as an effective approach in severely calcified iliofemoral disease [39]. Procedural success nevertheless remains dependent on careful anatomical selection, particularly with respect to vessel diameter, stenosis severity, and circumferential calcification [37,40]. Although these studies provide the principal evidence base for endovascular access facilitation techniques, their application to stenotic or heavily calcified abdominal aortic disease remains comparatively limited. 

Although angioplasty and intravascular lithotripsy have been increasingly used to facilitate transfemoral TAVI in patients with hostile iliofemoral anatomy, these strategies have been more extensively described and differ from the anatomical challenge encountered in hostile abdominal aortic anatomy. Accordingly, the present review focuses on interventions performed directly on the abdominal aorta, where published experience remains comparatively limited to eight case reports [26,28,31,43,44,45,46,47]. These reports encompass a heterogeneous spectrum of challenging aortic anatomies, including coral reef aorta, severe calcific stenosis of the distal abdominal aorta, infrarenal aortic stenosis, circumferential aortic calcification, and aortoiliac calcific disease. IVL represented the principal vessel-modification strategy across these reports, although the technique was adapted to the specific anatomical characteristics of each lesion. Morris et al. combined IVL with adjunctive balloon angioplasty for a coral reef aorta [26], whereas Fang et al. supplemented IVL with covered stent implantation in a severely calcified abdominal aorta [28]. Buonpane et al. incorporated intravascular ultrasound guidance and a kissing-balloon IVL technique to address extensive calcified infrarenal aortic disease [45]. Other reports employed IVL with stepwise low-pressure balloon inflations to increase vascular compliance and permit passage of large-bore delivery systems through severely calcified or stenotic abdominal aortic segments [31,43,44,46,47]. Despite differences in lesion morphology, adjunctive techniques, sheath sizes, and valve platforms, successful transfemoral valve delivery and implantation were reported in all eight cases, without major vascular complications. However, these favorable procedural outcomes derive exclusively from individual case reports and should be interpreted cautiously given the very small cumulative experience, heterogeneity of the treated anatomy, limited follow-up, and potential publication bias.

Available evidence suggests that endovascular modification of hostile abdominal aortic anatomy may permit transfemoral TAVI in carefully selected patients when conventional transfemoral access would otherwise be considered high risk or prohibitive. However, the evidence remains limited, consisting of only case reports, and does not establish the superiority or universal safety of this strategy over alternative access routes. Patient selection, detailed preprocedural imaging, individualized procedural planning, and appropriate bailout strategies are therefore essential, particularly when complex abdominal aortic anatomy is encountered.

Our case differed substantially from these reports because the principal obstacle to TF access was not calcific stenosis alone, but a fractured superior mesenteric artery stent fragment impacted within a severely atherosclerotic abdominal aortic stenosis. In this setting, conventional balloon angioplasty was selected as the primary endovascular strategy because it simultaneously expanded the stenotic segment and displaced the mobile stent fragment against the aortic wall, creating an adequate lumen for safe device passage. Although adjunctive IVL or implantation of an aortic stent or stent-graft could have been considered in the presence of circumferential calcification or persistent luminal compromise, neither was required after satisfactory angiographic and intraprocedural results were achieved. This case illustrates that endovascular optimization should be tailored to the underlying mechanism of access impairment and demonstrates that balloon angioplasty alone may be sufficient when the obstruction is predominantly mechanical rather than purely calcific. The present case expands this limited experience by demonstrating the feasibility of staged balloon angioplasty not only to enlarge a stenotic, heavily calcified abdominal aortic segment but also to stabilize a migrated mesenteric stent fragment against the aortic wall, thereby permitting subsequent passage of a large-bore transfemoral delivery system.

## 4. Limitations

This report has several limitations. First, the application of the technique described is limited to the specific conditions presented in our case report. Second, the literature review, while systematic in approach, was not exhaustive; the heterogeneity of the included studies (from isolated case reports to registries) precluded formal meta-analysis. Third, the two-year follow-up, while reassuring, represents a relatively short horizon for a structural cardiac intervention, and continued surveillance is required. A further limitation is the absence of dedicated vascular imaging during long-term follow-up; therefore, although no clinical symptoms of mesenteric or lower-limb ischemia were reported, the long-term anatomical stability of the apposed stent fragment and abdominal aortic intervention could not be objectively confirmed. Finally, the absence of IVL availability in our center at the time of the procedure influenced the choice of balloon angioplasty alone; centers with access to both modalities may approach similar cases differently.

## 5. Conclusions

Endovascular access facilitation may preserve the transfemoral approach to TAVI in carefully selected patients with hostile vascular anatomy. Although angioplasty and intravascular lithotripsy are well established for iliofemoral access preparation, experience with direct modification of the abdominal aorta remains limited to case reports. Available reports suggest the feasibility of IVL-based strategies for severely calcified or stenotic abdominal aortic disease; however, the small number of cases and potential publication bias preclude conclusions regarding their universal safety or superiority over alternative access routes. The present case highlights an additional and unusual access challenge—the combination of focal calcific abdominal aortic stenosis and a fractured, migrated SMA stent fragment—which was successfully managed by staged balloon angioplasty, simultaneously enlarging the aortic lumen and apposing the fragment against the aortic wall to permit subsequent transfemoral TAVI. Careful anatomical assessment, individualized procedural planning, and appropriate bailout strategies remain essential, while prospective data are needed to better define patient selection and outcomes for abdominal aortic access facilitation.

## Figures and Tables

**Table 1:** Published cases of superior mesenteric artery stent fracture. Abbreviations: CMI, chronic mesenteric ischemia; NS, not specified; PAD, peripheral artery disease; SMA, superior mesenteric artery; SMAA, superior mesenteric artery aneurysm.

	Author, Year	Patient	Context	Time to Fracture	Presentation	Fracture Pattern	Management	Outcome
1	Klepczyk et al., 2008 [19]	Adult (case 1)	CMI, SMA stenting	NS	Recurrent CMI symptoms	Stent fracture producing stenosis	NS	NS
		Adult (case 2)	CMI, SMA stenting	NS	Recurrent CMI symptoms	Stent fracture producing stenosis	NS	NS
2	Schellekens et al., 2011 [18]	Adult (case 1)	CMI, SMA stenting	NS	Recurrent CMI symptoms	Stent fracture producing stenosis	Reintervention with additional stent placement	Successful
		Adult (case 2)	CMI, SMA stenting	NS	Recurrent CMI symptoms	Stent fracture producing stenosis	Reintervention with additional stent placement	Successful
3	Canavati et al., 2013 [22]	82-year-old man	f-EVAR	1 year	Asymptomatic	Stent fracture and embolization	Reintervention with additional stent placement	Successful
		69-year-old man	f-EVAR	1 month	Asymptomatic	Stent fracture and embolization	Reintervention with additional stent placement	Successful
4	Juszkat et al., 2017 [20]	Adult	CMI, SMA stenting	7 months	Recurrent CMI symptoms	Stent fracture with stent displacement	Reintervention with additional stent placement	Successful
5	Robins et al., 2019 [21]	69-year-old woman	CMI, SMA stenting	NS	Recurrent CMI symptoms	Recurrent SMA stent fracture	NS	NS
6	Bontinis et al., 2022 [23]	56-year-old man	CMI, PAD	NS	Persistent postprandial discomfort; abdominal X-ray raised suspicion	Type III stent fracture (Allie classification)	Reintervention with additional stent placement	Successful
7	He Y. et al., 2024 [24]	62-year-old	SMAA, SMA stenting	2 years	Recurrent symptoms	Stent fracture producing stenosis	Surgery	Successful

**Table 2:** Published Studies on Endovascular Management of (A) Iliofemoral Disease and (B) Hostile Abdominal Aortic Anatomy to Facilitate Transfemoral TAVI. Abbreviations: AA, alternative access; AE, adverse events; BA, balloon angioplasty; BEV, balloon-expandable valve; BT, blood transfusions; IVL, intravascular lithotripsy; NS, not specified; PVI, peripheral vascular intervention; SEV, self-expandable valve; SMA, superior mesenteric artery; TAVI, transcatheter aortic valve implantation; TAX, transaxillary; TF, transfemoral.

	Author, Year	Study Type	Mean Age	Target Vessel/Aortic Pathology	Procedural Strategy	Technique	Sheath/ Valve	Technical Success	Complications	Outcome/ Follow-Up
A. Iliofemoral access-facilitation studies—supporting evidence for endovascular vessel preparation										
1	Staniloae et al. 2019 [33]	Case series (*n* = 28)	82.8 ± 8.6 years	Iliofemoral	Planned access facilitation	Preparatory PTA (*n* = 12); orbital atherectomy + PTA (*n* = 1)	16F/18-Fr sheath; SEV	92.9%	Major bleeding complication 3.5% Major vascular complications 7.1%	NS
2	Sawaya et al. 2021 [2]	Prospective single-center (*n* = 50)	78.3 ± 6.7 years	Iliofemoral	Planned access facilitation	IVL 7.0 × 60 mm + PTA (92%)	14/16-Fr sheath; BEV	100%	None	NS
3	Nardi et al. 2022 [32]	Multicenter prospective observational (*n* = 108)	80.5 ± 6.2 years	Iliofemoral	Mixed (planned + bailout)	IVL (7.0 mm Shockwave); predilatation 5–6 mm in 17.6%	16F/18-Fr sheath; SEV	100%	Major access site complications 1.8% AKI 6.5%	In-hospital mortality 2.8%
4	Linder et al. 2024 [34]	Comparative (IVL-TAVI vs. TAX-TAVI) (*n*_1_ = 30 vs. *n*_2_ = 44)	78.2 ± 8.6 years	Iliofemoral	Planned access facilitation	IVL (8 × 60 mm, 210 pulses)	14/16-Fr sheath; BEV	93.3% (28/30) vs. 81.8% (36/44)	Major access site complications 3.3% IVL-TAVI vs. 20.5% TAX-TAVI; *p* = 0.042	30-day mortality 10% IVL-TAVI vs. 31.8% TAX-TAVI
5	Bansal et al. 2021 [35]	Retrospective US database (NIS); TAVI (*n* = 99,654) TAVI + PVI (*n* = 4397) TF-TAVI + PVI *n* = 2723 AA-TAVI (*n* = 2538)	80.2 ± 8.3 years	Iliofemoral	Mixed (planned + bailout)	PVI (various: PTA, stenting, atherectomy) includes bailout PVI	NS	NS	TAVI + PVI vs. TAVI -AKI 17.6% vs. 10.8% -BT 16.0% vs. 11.3% TF-TAVI + PVI vs. AA-TAVI -AKI 14.3% vs. 22.7% -BT 13.3% vs. 13.4%	30-day mortality TAVI + PVI vs. TAVI 4.2% vs. 1.5% TF-TAVI + PVI vs. AA-TAVI 3.0% vs. 4.6%
6	Isogai et al. 2023 [36]	Retrospective single-center (*n* = 2246; *n*_1_ = 136 PVI)	79.1 ± 9.3 years	Iliofemoral	Mixed (planned + bailout)	PVI (various: PTA, stenting, atherectomy) includes bailout PVI	14/16-Fr sheath; BEV	98%	In hospital death: 0.0% (TF + PVI) vs. 0.2% (TF no-PVI) vs. 0.7% (non-TF) In hospital MACCE: 0.0% (TF + PVI) vs. 0.8% (TF no-PVI) vs. 4.3% (non-TF)	2-year mortality 15.4% (TF + PVI) vs. 20.7% (TF no-PVI) vs. 40.7% (non-TF) 2-year MACCE: 16.9% (TF + PVI) vs. 23.0% (TF no-PVI) vs. 45.0% (non-TF)
7	Tom et al. 2025 [37]	Retrospective single-center (*n* = 92 IVL/2862 TAVR)	78 ± 9.2 years	Iliofemoral	Planned access facilitation	IVL (7-mm balloon in 73.9%)	16F/18-Fr sheath; SEV 14/16-Fr sheath; BEV	100%	Major vascular complications 1.1% Minor vascular complications 6.5%	30-day mortality 1.1%
8	Imran et al. 2024 [42]	Retrospective US (*n* = 129,655 TAVR; 1242 IVL)	78.4 ± 8.9 years	Iliofemoral	Mixed (planned + bailout)	IVL (Shockwave)	16F/18-Fr sheath; SEV 14/16-Fr sheath; BEV	99.7% TF completion	Primary composite (in-hospital all-cause death, stroke, vascular complication, need for surgical vascular intervention, and major bleeding): 21.9% (IVL) vs. 13.7% (non-IVL), *p* < 0.001;	30-day mortality 1.9% IVL vs. 1.4% non-IVL
9	von Zur Mühlen et al. 2025 [38]	Single-center prospective + retrospective (*n* = 1229; 26 IVL prospective)	83 (78–86) years	Iliofemoral	Planned access facilitation	IVL (Shockwave)	16F/18-Fr sheath; SEV 14/16-Fr sheath; BEV	100%	Major vascular complications 7.7% IVL vs. 4.4% no-IVL	3-month mortality 0% IVL vs. 6.0% no-IVL
10	Belkin et al. 2025 [39]	Multicenter (Hostile TAVI Registry; *n* = 518: 352 PTA vs. 166 IVL)	81.1 ± 6.4 years	Iliofemoral	Planned access facilitation	67.9% PTA 32.1% IVL	16F/18-Fr sheath; SEV 14/16-Fr sheath; BEV	100%	Major bleeding 14.0% PTA vs. 7.0% IVL Major vascular complications 21.7% PTA vs. 13.3% IVL	3-year mortality 34.9% PTA vs. 38.6% IVL
11	Del Sole et al. 2024 [40]	Case series (*n* = 13)	80.8 ± 6.9 years	Iliofemoral	Bail-out	IVL performed after PTA failure	14-Fr sheath; SEV 14/16/18-Fr sheath; BEV	92.3%	Major bleeding 7.70%	3-year mortality 19.2%
12	Mustafa et al. 2026 [41]	Comparative (*n* = 150 PAD) 24 IVL TF- TAVI vs. 126 AA-TAVI	79.9 ± 7.5 years	Iliofemoral	Planned access facilitation	Facilitated (IVL) TF-TAVI vs. AA-TAVI	16F/18-Fr sheath; SEV 14/16-Fr sheath; BEV	100%	Primary composite: 8% IVL TF-TAVI vs. 14% AA-TAVI	1-month mortality 0% IVL TF-TAVI vs. 2% AA-TAVI
B. Abdominal aortic interventions—directly relevant evidence										
1	Morris et al. 2021 [26]	Case report	70-year-old female	Coral reef aorta (suprarenal/infrarenal)	Planned access facilitation	IVL M5 7.0 mm + BA 10 × 60 mm	18-Fr sheath; 26-mm SEV	100%	None	Discharged; good clinical outcome at short-term follow-up
2	Ponna et al. 2023 [43]	Case report	84-year-old female	Severe calcific stenosis of distal abdominal aorta	Planned access facilitation	IVL M5 7.0 mm Shockwave; stepwise low-pressure inflations	18-Fr sheath; 23-mm SEV	100%	None	Discharged next day; uneventful recovery
3	Fang et al. 2025 [28]	Case report	84-year-old female	Severely calcified abdominal aorta	Planned access facilitation	IVL M5 7.0 mm Shockwave; 18-mm ovation covered stent	14-Fr E-sheath; 23-mm BEV	100%	None	Discharged next day; uneventful recovery
4	Nishi et al. 2026 [31]	Case report	84-year-old female	Severely calcified abdominal aorta	Planned access facilitation	IVL M5 7.0 mm Shockwave; stepwise low-pressure inflations	18-Fr Gore DrySeal sheath; 23-mm SEV	100%	None	Same-day discharge; uneventful recovery
5	Buonpane et al. 2024 [45]	Case report	84-year-old male	Extensive calcified atherosclerosis of the infrarenal abdominal aorta	Planned access facilitation	IVUS, IVL 7.0 mm; Kissing Shockwave Balloon Technique	14-Fr E-sheath; 23-mm BEV	100%	None	Discharged 3rd day; uneventful recovery
6	Al Jabri et al. 2025 [46]	Case report	71-year-old male	Infrarenal abdominal aortic stenosis	Planned access facilitation	IVL L6 12.0 × 30 mm Shockwave; stepwise low-pressure inflations	65-cm GORE^®^ DRYSEAL Flex Introducer Sheath 18-Fr 26-mm SEV	100%	None	Same-day discharge; uneventful recovery
7	Rahimov et al. 2025 [47]	Case report	73-year-old male	Circumferential calcification of aorta and iliofemoral arteries	Planned access facilitation	IVL M5 7.0 mm Shockwave; stepwise low-pressure inflations	16-Fr E-sheath; 29-mm BEV	100%	None	Discharged on day 3; uneventful recovery
8	Bhastana et al. 2025 [44]	Case report	76-year-old female	Aortoiliac calcific stenosis	Planned access facilitation	IVL M5 7.0 mm Shockwave; stepwise low-pressure inflations	14-Fr sheath; 23-mm SEV	100%	None	Same-day discharge; uneventful recovery

## Data Availability

The data presented are contained within the article. Additional patient-specific clinical and imaging data are not publicly available to protect patient privacy and confidentiality.

## References

[1] Carroll J.D., Mack M.J., Vemulapalli S., Herrmann H.C., Gleason T.G., Hanzel G., Deeb G.M., Thourani V.H., Cohen D.J., Desai N. (2020). STS-ACC TVT Registry of Transcatheter Aortic Valve Replacement. J. Am. Coll. Cardiol..

[2] Sawaya F.J., Bajoras V., Vanhaverbeke M., Wang C., Bieliauskas G., Søndergaard L., De Backer O. (2021). Intravascular Lithotripsy-Assisted Transfemoral TAVI: The Copenhagen Experience and Literature Review. Front. Cardiovasc. Med..

[3] Blanke P., Weir-McCall J.R., Achenbach S., Delgado V., Hausleiter J., Jilaihawi H., Marwan M., Nørgaard B.L., Piazza N., Schoenhagen P. (2019). Computed Tomography Imaging in the Context of Transcatheter Aortic Valve Implantation (TAVI)/Transcatheter Aortic Valve Replacement (TAVR): An Expert Consensus Document of the Society of Cardiovascular Computed Tomography. JACC Cardiovasc. Imaging.

[4] Perrin N., Bonnet G., Leroux L., Ibrahim R., Modine T., Ben Ali W. (2021). Transcatheter Aortic Valve Implantation: All Transfemoral? Update on Peripheral Vascular Access and Closure. Front. Cardiovasc. Med..

[5] Cerillo A.G., Voetsch A., Michel J., Ruge H. (2022). Editorial: Transcatheter Aortic Valve Implantation: All Transfemoral? Update on Vascular Access and Closure. Front. Cardiovasc. Med..

[6] Praz F., Borger M.A., Lanz J., Marin-Cuartas M., Abreu A., Adamo M., Ajmone Marsan N., Barili F., Bonaros N., Cosyns B. (2025). 2025 ESC/EACTS Guidelines for the Management of Valvular Heart Disease: Developed by the Task Force for the Management of Valvular Heart Disease of the European Society of Cardiology (ESC) and the European Association for Cardio-Thoracic Surgery (EACTS). Eur. Heart J..

[7] Otto C.M., Nishimura R.A., Bonow R.O., Carabello B.A., Erwin J.P., Gentile F., Jneid H., Krieger E.V., Mack M., McLeod C. (2021). 2020 ACC/AHA Guideline for the Management of Patients with Valvular Heart Disease: A Report of the American College of Cardiology/American Heart Association Joint Committee on Clinical Practice Guidelines. Circulation.

[8] Petronio A.S., De Carlo M., Bedogni F., Maisano F., Ettori F., Klugmann S., Poli A., Marzocchi A., Santoro G., Napodano M. (2012). 2-Year Results of CoreValve Implantation through the Subclavian Access: A Propensity-Matched Comparison with the Femoral Access. J. Am. Coll. Cardiol..

[9] Kindzelski B., Mick S.L., Krishnaswamy A., Kapadia S.R., Attia T., Hodges K., Siddiqi S., Lowry A.M., Blackstone E.H., Popovic Z. (2021). Evolution of Alternative-Access Transcatheter Aortic Valve Replacement. Ann. Thorac. Surg..

[10] Corcione N., Giordano S., Ferraro P., Morello A., Cimmino M., Albanese M., Avellino R., Biondi-Zoccai G., Pepe M., Giordano A. (2026). Percutaneous Axillary vs. Femoral Access for Transcatheter Aortic Valve Replacement: Insights from a 2164-Patient Study. Int. J. Cardiol..

[11] Wee I.J.Y., Stonier T., Harrison M., Choong A.M.T.L. (2018). Transcarotid Transcatheter Aortic Valve Implantation: A Systematic Review. J. Cardiol..

[12] Meertens M.M., Adam M., Beckmann A., Ensminger S., Frerker C., Seiffert M., Sinning J.M., Bekeredjian R., Walther T., Beyersdorf F. (2025). Non-Femoral Focused Transaxillary Access in TAVI: GARY Data Analysis and Future Trends. Clin. Res. Cardiol..

[13] Chidester J., Donisan T., Desai P.V., Banthiya S., Zaghloul A., Jessen M.E., Park K., Tan W., Tsai S., Huffman L. (2025). Access Options for Transcatheter Aortic Valve Replacement. J. Clin. Med..

[14] Panchal H.B., Ladia V., Amin P., Patel P., Veeranki S.P., Albalbissi K., Paul T. (2014). A Meta-Analysis of Mortality and Major Adverse Cardiovascular and Cerebrovascular Events in Patients Undergoing Transfemoral versus Transapical Transcatheter Aortic Valve Implantation Using Edwards Valve for Severe Aortic Stenosis. Am. J. Cardiol..

[15] Lederman R.J., Babaliaros V.C., Lisko J.C., Rogers T., Mahoney P., Foerst J.R., Depta J.P., Muhammad K.I., McCabe J.M., Pop A. (2022). Transcaval versus Transaxillary TAVR in Contemporary Practice: A Propensity-Weighted Analysis. JACC Cardiovasc. Interv..

[16] Antiochos P., Kirsch M., Monney P., Tzimas G., Meier D., Fournier S., Ferlay C., Nowacka A., Roffi M., Muller O. (2024). Transcaval versus Supra-Aortic Vascular Accesses for Transcatheter Aortic Valve Replacement: A Systematic Review with Meta-Analysis. J. Clin. Med..

[17] Touma G., Fairley S., Ada C., Wong B., Khialani B. (2025). A Contemporary Framework for the Diagnosis and Management of Stent Fracture. Catheter. Cardiovasc. Interv..

[18] Schellekens J.F., Vos J.A., van den Heuvel D.A.F., Boersma D., de Vries J.P.P.M. (2011). Superior Mesenteric Artery Stent Fracture Leading to Recurrent Mesenteric Ischemia. Vasc. Endovasc. Surg..

[19] Klepczyk L., Keeling W.B., Stone P.A., Shames M.L. (2008). Superior Mesenteric Artery Stent Fracture Producing Stenosis and Recurrent Chronic Mesenteric Ischemia: Case Report. Vasc. Endovasc. Surg..

[20] Juszkat R., Klimont M., Śliwa M., Krasiński Z. (2017). Fractured Superior Mesenteric Artery Stent with Stent Displacement Leading to Recurrent Symptoms of Superior Mesenteric Ischemia. Vasc. Endovasc. Surg..

[21] Robins J.E., Young Z., Glocker R.J., Stoner M.C., Doyle A.J. (2019). Recurrent Superior Mesenteric Artery Stent Fracture. Ann. Vasc. Surg..

[22] Canavati R., How T.V., Brennan J.A., Vallabhaneni S.R., Fisher R.K., McWilliams R.G. (2013). Fractured Superior Mesenteric Artery Stents after Fenestrated Endovascular Aneurysm Repair. J. Vasc. Surg..

[23] Bontinis V., Koutsoumpelis A., Chatziantoniou G., Rafailidis V., Papachristodoulou A., Ktenidis K. (2022). Superior Mesenteric Artery Stent Fracture: A Case Report. Hell. J. Vasc. Endovasc. Surg..

[24] He Y., Zhang J., Li G., Zhou H. (2024). Treatment of Stent Rupture after Endovascular Treatment of Superior Mesenteric Aneurysm with Open Surgery. Vascular.

[25] Tallarita T., Tenorio E.R., Kärkkäinen J.M., Oderich G.S., Dryjski M.L., Harris L.M. (2022). Complications in Angioplasty and Stenting of Mesenteric and Renal Artery Disease. Complications in Endovascular Surgery: Peri-Procedural Prevention and Treatment.

[26] Morris J.P., Cadogan D., Casserly I.P. (2021). Intravascular Lithotripsy-Assisted Balloon Angioplasty to Facilitate Transfemoral Transcatheter Aortic Valve Implantation in a Patient with Coral Reef Aorta. BMJ Case Rep..

[27] Senguttuvan N.B., Ellozy S., Tejani F., Kovacic J., Kini A.S., Sharma S.K., Dangas G.D. (2015). TAVR through Heavily Calcified Aorta Following Atheroma Retrieval with the “Elevator” Technique. J. Invasive Cardiol..

[28] Fang J.X., O’Neill B.P., Frisoli T.M., Lee J.C., Giustino G., Engel Gonzalez P., O’Neill W.W., Villablanca P.A. (2025). Aortic Valve Stenosis and Calcified Abdominal Aortic Stenosis Treated with Lithotripsy-Facilitated TAVR and Aortic Stenting. JACC Case Rep..

[29] Naganuma T., Kawamoto H., Onishi H., Nakamura S. (2018). Successful Rotational Atherectomy for Abdominal Aortic Stenosis with Severe Calcification in a Patient Treated with Transfemoral Transcatheter Aortic Valve Implantation. Kardiol. Pol..

[30] Hallisey M.J., Meranze S.G., Parker B.C., Rholl K.S., Miller W.J., Katzen B.T., van Breda A. (1994). Percutaneous Transluminal Angioplasty of the Abdominal Aorta. J. Vasc. Interv. Radiol..

[31] Nishi T., Kalavakunta J.K., Sood A., Pratt J.W., Saltiel F., Gupta V. (2026). Intravascular Lithotripsy of Negatively Remodeled Calcified Abdominal Aorta to Facilitate Transfemoral Transcatheter Aortic Valve Replacement. JACC Case Rep..

[32] Nardi G., De Backer O., Saia F., Søndergaard L., Ristalli F., Meucci F., Stolcova M., Mattesini A., Demola P., Wang X. (2022). Peripheral Intravascular Lithotripsy for Transcatheter Aortic Valve Implantation: A Multicentre Observational Study. EuroIntervention.

[33] Staniloae C.S., Jilaihawi H., Amoroso N.S., Ibrahim H., Hisamoto K., Sin D.N., Lee H., Du R., Zhao Z.-G., Neuburger P.J. (2019). Systematic Transfemoral Transarterial Transcatheter Aortic Valve Replacement in Hostile Vascular Access. Struct. Heart.

[34] Linder M., Grundmann D., Kellner C., Demal T., Waldschmidt L., Bhadra O., Ludwig S., Voigtländer L., von der Heide I., Nebel N. (2024). Intravascular Lithotripsy-Assisted Transfemoral Transcatheter Aortic Valve Implantation in Patients with Severe Iliofemoral Calcifications: Expanding Transfemoral Indications. J. Clin. Med..

[35] Bansal A., Kalra A., Kumar A., Campbell J., Krishnaswamy A., Kapadia S.R., Reed G.W. (2021). Outcomes of Combined Transcatheter Aortic Valve Replacement and Peripheral Vascular Intervention in the United States. JACC Cardiovasc. Interv..

[36] Isogai T., Agrawal A., Shekhar S., Spilias N., Puri R., Krishnaswamy A., Unai S., Yun J.J., Kapadia S.R., Reed G.W. (2023). Comparison of Outcomes Following Transcatheter Aortic Valve Replacement Requiring Peripheral Vascular Intervention or Alternative Access. J. Am. Heart Assoc..

[37] Tom S., Tully A., Kikuchi Y., Crawford K., Binongo J., Wei J.W., Gleason P., Xie J., Devireddy C.M., Grubb K.J. (2025). Peripheral Intravascular Lithotripsy to Facilitate Transfemoral Transcatheter Aortic Valve Replacement—Defining Optimal Treatable Peripheral Arterial Disease Burden. Cardiovasc. Revasc. Med..

[38] von zur Mühlen C., Valina C., Vagner L., Mack L., Asmussen A., Lindau A., Ruile P., Hein M., Moser M., Stachon P. (2025). Intravascular Lithotripsy Enables Safe and Effective Transfemoral Aortic Valve Replacement. Clin. Res. Cardiol..

[39] Belkin D., Bental T., Palmerini T., Kornowski R., Codner P. (2025). Standard Percutaneous Transluminal Angioplasty versus Intravascular Lithotripsy to Facilitate Transfemoral Transcatheter Aortic Valve Implantation in Patients with Aortic Stenosis and Severe Peripheral Arterial Disease. J. Clin. Med..

[40] Del Sole P.A., Lunardi M., Andreaggi S., Fezzi S., Pesarini G., Scarsini R., Ribichini F. (2024). Intravascular Lithotripsy-Assisted Transfemoral Transcatheter Aortic Valve Implantation after Failed Balloon Angioplasty in Patients with Severe Calcified Peripheral Artery Disease. J. Invasive Cardiol..

[41] Mustafa A., Wang D., Dutton C., Kodra A., Shakir M., Goldberg Y., Chau M., Thampi S., Mehla P., Wei C. (2026). Early Outcomes of Facilitated Transfemoral versus Alternative Access for Transcatheter Aortic Valve Replacement in Patients with Peripheral Arterial Disease. J. Invasive Cardiol..

[42] Imran H.M., Has P., Kassis N., Shippey E., Elkaryoni A., Gordon P.C., Sharaf B.L., Soukas P.A., Hyder O.N., Sellke F. (2024). Characteristics, Trends, and Outcomes of Intravascular Lithotripsy-Assisted Transfemoral Transcatheter Aortic Valve Replacement in the United States. JACC Cardiovasc. Interv..

[43] Ponna P.K., Gonuguntla A., Botta R.K., Fischell T., Agrawal Y. (2023). Shockwave Lithotripsy of Calcific Stenosis of the Distal Abdominal Aorta to Facilitate Transcatheter Aortic Valve Replacement. Cardiovasc. Revasc. Med..

[44] Bhastana V.J., Bharadi S., Menon R., Kapadiya A. (2025). Peripheral Intravascular Lithotripsy in Transcatheter Aortic Valve Implantation: A Case Report. Eur. Heart J. Case Rep..

[45] Buonpane A., Trimarchi G., Palmieri C., Al Jabri A.A.A., Berti S., Rizza A. (2024). Kissing Shockwave Balloon in a Case of Extensive Calcified Abdominal Aorta during Transfemoral TAVI. Curr. Probl. Cardiol..

[46] Al Jabri A., Ravani M., Trianni G., Gasbarri T., Casula M., Berti S. (2025). Transfemoral TAVI in a High-Risk Patient with Porcelain Aorta and Severe Subrenal Abdominal Aortic Stenosis: A Case Report. J. Cardiovasc. Dev. Dis..

[47] Rahimov U., Zeynalov R., Karimli E., Aliyev F., Hajiyev E., Abdulalimova K., Mustafaeva S., Kilic T. (2025). Shockwave Lithotripsy-Assisted TAVI in a Patient with Severely Calcified Peripheral Arteries and Porcelain Aorta. Egypt. Heart J..

